# Hemodynamic and Structural Comparison of Human Fetal Heart Development Between Normally Growing and Hypoplastic Left Heart Syndrome-Diagnosed Hearts

**DOI:** 10.3389/fphys.2022.856879

**Published:** 2022-03-23

**Authors:** Huseyin Enes Salman, Reema Yousef Kamal, Ziyad M. Hijazi, Huseyin Cagatay Yalcin

**Affiliations:** ^1^ Department of Mechanical Engineering, TOBB University of Economics and Technology, Ankara, Turkey; ^2^ Pediatric Cardiology Division, Hamad General Hospital, Hamad Medical Corporation, Doha, Qatar; ^3^ Sidra Heart Center, Sidra Medicine, Weill Cornell Medical College, Doha, Qatar; ^4^ Biomedical Research Center, Qatar University, Doha, Qatar

**Keywords:** congenital heart defects, fetal heart development, hypoplastic left heart syndrome, computational fluid dynamics, tricuspid valve, mitral valve, disturbed hemodynamics, echocardiography

## Abstract

Congenital heart defects (CHDs) affect a wide range of societies with an incidence rate of 1.0–1.2%. These defects initiate at the early developmental stage and result in critical health disorders. Although genetic factors play a role in the formation of CHDs, the occurrence of cases in families with no history of CHDs suggests that mechanobiological forces may also play a role in the initiation and progression of CHDs. Hypoplastic left heart syndrome (HLHS) is a critical CHD, which is responsible for 25–40% of all prenatal cardiac deaths. The comparison of healthy and HLHS hearts helps in understanding the main hemodynamic differences related to HLHS. Echocardiography is the most common imaging modality utilized for fetal cardiac assessment. In this study, we utilized echocardiographic images to compare healthy and HLHS human fetal hearts for determining the differences in terms of heart chamber dimensions, valvular flow rates, and hemodynamics. The cross-sectional areas of chamber dimensions are determined from 2D b-mode ultrasound images. Valvular flow rates are measured via Doppler echocardiography, and hemodynamic quantifications are performed with the use of computational fluid dynamics (CFD) simulations. The obtained results indicate that cross-sectional areas of the left and right sides of the heart are similar for healthy fetuses during gestational development. The left side of HLHS heart is underdeveloped, and as a result, the hemodynamic parameters such as flow velocity, pressure, and wall shear stress (WSS) are significantly altered compared to those of healthy hearts.

## Introduction

With an incidence rate of 1.0–1.2 percent, congenital heart defects (CHDs) impact a wide variety of societies across the world (Hoffman, 2013). The formation of CHDs begins in the early stages of fetal heart development and involves the incorrect formation of the ventricles, arteries, and valves (Hoffman and Kaplan, 2002; Salman and Yalcin, 2021). CHDs’ etiology and causation still remain unclear (Yalcin et al., 2011). Despite the fact that genetic factors play a significant role in the development of CHDs (Jarrell et al., 2019; Dyer and Rugonyi, 2021), they are not the only cause of early cardiac disorders (Zaidi et al., 2013; Rugonyi, 2016). For newborns, only 2–4% of CHDs are reported in the families with a history of CHDs, indicating that the majority of cases occur in newborns with no family history of early cardiac disorders (Øyen et al., 2009).

One of the most serious CHDs is known as hypoplastic left heart syndrome (HLHS), in which the left ventricle is underdeveloped and unable to maintain systemic circulation in the cardiovascular system (Norwood, 1991; Mäkikallio et al., 2006; Barron et al., 2009). HLHS is responsible for 25–40% of all prenatal cardiac deaths, and it is generally diagnosed during the second trimester of pregnancy by utilizing fetal echocardiogram, electrocardiogram, or cardiac MRI (Saraf et al., 2019). While some genetic factors have been shown to contribute HLHS, in most cases, gene mutations cannot explain the initiation and progression of HLHS. Disturbed hemodynamics has been suggested as an important factor for such cases (Salman and Yalcin, 2021). Compared to healthy fetal hearts, the hemodynamic environment of HLHS hearts shows substantial differences due to the underdevelopment of the left heart (Salman et al., 2021a), which results in a significantly deteriorated life quality for the patients with HLHS (Dempster et al., 2017). The blood flow in HLHS hearts is disturbed as a consequence of the developmental disorders. As a result, the heart’s shear stress distribution and hemodynamics are seriously altered compared to the healthy fetal hearts (Zebhi et al., 2020).

To fully comprehend the distinctions between the healthy and HLHS hearts, the clinical data should be elucidated to quantify the geometric changes, valvular flow rates, and hemodynamics inside the heart chambers during the gestational stages. Nevertheless, clinical *in vivo* flow measurements, three-dimensional (3D) modeling (Rykiel et al., 2020), and hemodynamic quantifications (Courchaine et al., 2018) are difficult tasks due to the small dimensions and highly dynamic nature of the fetal hearts (Chen et al., 2017). *In vivo* flow velocities can only be resolved at certain planes inside the heart chambers and arteries using Doppler echocardiography, which is an ultrasound-based imaging modality (Yalcin et al., 2002; Benslimane et al., 2019). Therefore, an appropriate modality is required to reveal the complex hemodynamic features within the entire heart by utilizing the *in vivo* flow measurements.

At that point, the use of computational fluid dynamics (CFD) modeling is a beneficial tool to explore the hemodynamics inside the complicated geometries such as a growing human fetal heart (Marsden and Feinstein, 2015; Courchaine and Rugonyi, 2018). The advantage of CFD modeling is enabling to investigate the hemodynamics in an isolated region inside the heart by employing physiologically measured realistic inflow conditions (Courchaine et al., 2019; Salman et al., 2021b). The CFD modeling approach is widely used to investigate the complex flow fields in the cardiovascular system (Yalcin et al., 2017; Salman et al., 2019; Bäumler et al., 2020; Salman and Yalcin, 2020; Mutlu et al., 2021). On the other hand, only few research studies have been published in the literature that employ CFD modeling to elucidate the flow patterns in human embryonic hearts (DeGroff et al., 2003; Pennati et al., 2008; Saw et al., 2017; Wiputra et al., 2018). In several computational modeling studies, the volumetric contractions of the left (Lai et al., 2016) and right ventricles (Wiputra et al., 2016; Zebhi et al., 2020) are analyzed to determine the flow characteristics in human fetal hearts. Furthermore, hemodynamic evaluations and computational models for the human fetal hearts can provide a thorough knowledge of CHDs, particularly for demystifying the mechanobiological pathways of HLHS development. We have recently utilized the CFD methodology outlined here, to examine the evolving cardiac hemodynamics in normally developing human fetal hearts (Salman et al., 2021b).

As a follow-up to our previous work, in this study, HLHS human hearts are compared to healthy human hearts at the fetal development stages in order to determine the main differences and clues about the etiology and growth of HLHS. For this purpose, three different measurements are adapted for the comprehensive comparison of HLHS and healthy hearts, which are the comparison of the cross-sectional areas of heart chambers, the comparison of the flow rates at mitral and tricuspid valves, and the comparison of hemodynamics using CFD simulations.

## Materials and Methods

Three different measurements are employed for the comparison of the control (healthy) and HLHS hearts. In the first approach, the cross-sectional ultrasound images of fetal hearts are used to determine the heart chamber areas at various gestational stages. Obtaining 3D volumes directly was not possible; instead we measured 2D cross sectional areas that would represent 3D volumes. Secondly, blood flow rates at the mitral and tricuspid valves are measured using Doppler echocardiography to observe the differences between the HLHS and control hearts at different developmental stages. In the third method, the CFD modeling approach is employed for analyzing the hemodynamic differences between the control and HLHS hearts. CFD simulations mimic the *in vivo* flow conditions inside the fetal heart geometries by employing physiological boundary conditions and solving physically governing flow equations.

### Cross-Sectional Areas of Fetal Heart Chambers

In this section, heart chamber areas are analyzed for human fetuses by comparing the healthy and HLHS hearts. The cross-sectional areas of the left ventricle (LV), left atria (LA), right ventricle (RV), and right atria (RA) are determined at the instant of the highest LV volume at diastole. The borders of the heart chambers are determined and the bounded areas are calculated using the ANSYS DesignModeler (Canonsburg, PA, United States) software package. For the analysis, 14 control and 16 HLHS fetal hearts are employed at different gestational stages given in Table 1.

**TABLE 1:** The number of sample fetal hearts used for analysis of chamber cross-sectional areas at different gestational stages.

Gestational stage	Number of HLHS hearts	Number of control hearts
Week 16	—	2
Week 17	—	2
Week 19	—	2
Week 22	1	—
Week 23	2	1
Week 24	3	3
Week 25	1	1
Week 26	—	1
Week 27	1	1
Week 28	3	—
Week 31	2	1
Week 32	2	—
Week 34	1	—

### Flow Measurements at Mitral and Tricuspid Valves

The blood flow waveforms are determined by measuring the velocities at mitral and tricuspid valves of the control and HLHS hearts using echocardiography, which is a method that examines the flow velocities in the heart by utilizing the Doppler effect (Benslimane et al., 2019). In this method, high-frequency sound waves are used to determine the speed and direction of blood flow at specific locations in the heart (Wang et al., 2018). The flow measurements are performed for one complete cardiac cycle. The sample hearts provided in Table 1 which consists of 14 control and 16 HLHS fetal hearts are used for flow measurements in the mitral and tricuspid valves.

### Computational Fluid Dynamics Simulations

CFD analysis is a numerical method based on discretizing complex geometry and solving the physically governing Navier–Stokes equations in order to determine the flow variables such as velocity, pressure, and WSS. The Navier–Stokes equations are provided in Eqs 1, 2, where v denotes the velocity vector, ρ denotes the mass density of the fluid, τ denotes the tensor of fluid stress, and f denotes the body forces such as the gravitational force. The effect of gravity is negligible on the interested results; therefore, the body forces are assumed to be zero.

∇⋅v=0, (1)

$$\rho \frac{\partial v}{\partial t}+\rho \left(v\right)\cdot \nabla v-\nabla \cdot \tau =f.$$ (2)

**FIGURE 1:**
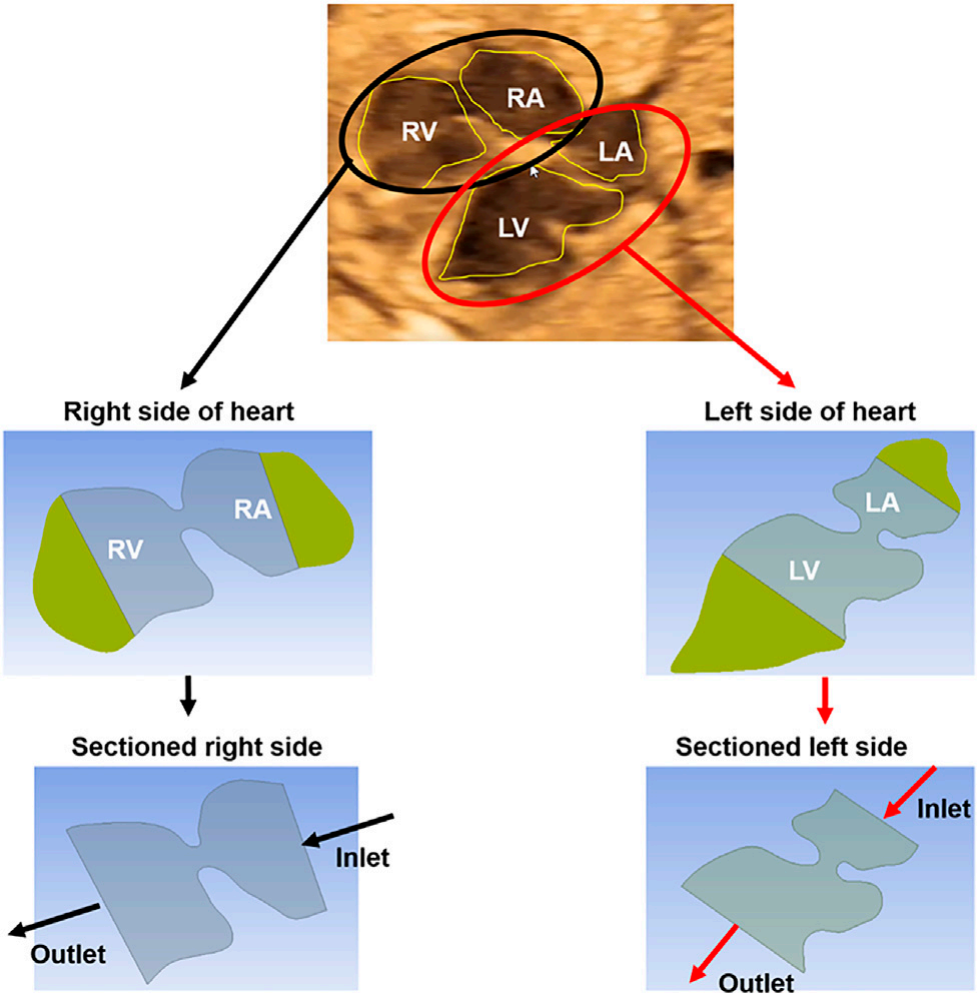
The methodology of CFD analysis. A sample ultrasound image of a 23-week control heart is used to demonstrate the methodology. First, the ultrasound-based geometric models are generated by determining the borders of the heart chambers. The left and right sides of the hearts are modeled and analyzed separately. The heart chambers are sectioned to apply the boundary conditions at the inlet and outlet of the fetal heart models. Inlet flow profiles are adjusted up to reaching the clinical velocity levels at the mitral and tricuspid valves which are measured *via* Doppler echocardiography. The mitral and tricuspid valves are in the left and right sides of the heart, respectively.

Two-dimensional (2D) ultrasound images are used to prepare the healthy and HLHS geometries. The left and right sides of the hearts are analyzed separately. In order to apply the physical boundary conditions, about one-third of the chamber areas are sectioned as shown in Figure 1. Then, the inlet flow velocities are applied on the sectioned edges of the atrium considering the clinically measured valve flow rates. Except the inlet and outlet, the boundaries of the sectioned heart chambers are modeled as static walls with no displacement and velocity. The no-slip boundary condition is applied on the walls to have zero flow velocity on the wall boundaries. The outlets of the flow domain are set with zero pressure. Due to the static wall assumption, CFD simulations are considered to be accurate only during the left ventricular filling phase, where the mitral valve is completely open and the valve-neighboring regions in the heart are relatively stagnant. The instants other than the left ventricular filling phase are not in the interest of this study. The heat transfer is neglected in the simulations due to the indiscernible effect on the investigated flow parameters.

For each fetal heart, unique flow profiles are applied at the inlet boundaries of the CFD models. Inlet flow velocities are adjusted until reaching the clinically measured flow velocities at the mitral and tricuspid valves. Therefore, the velocities predicted by the CFD simulations are validated and guaranteed to be the same with the clinical measurements at the valve proximity. For the CFD simulations, the methodology used in this study is the same with a previously published article of our research group (Salman et al., 2021b). One control heart and one HLHS heart are investigated at gestational weeks of 23, 27, and 31.

CFD simulations are performed using 20 sequential time steps with 0.025s increments. The blood is modeled as a homogeneous Newtonian fluid with a viscosity of 3.5 cP (Roldán-Alzate et al., 2015) and a mass density of 1060 kg/m^3^ (Bove et al., 2003). ANSYS Fluent (Canonsburg, PA, United States) finite element software package is used to perform CFD simulations using an incompressible pressure-based flow solver with laminar flow assumption. The pressure-based solver involves a process referred to as the projection method, where the mass conservation in the flow field is achieved by solving a pressure equation that is obtained from the continuity and momentum equations. In this method, the velocity field which is corrected by the pressure, satisfies the continuity of the flow. This solution procedure requires iterations, in which the complete set of governing equations is solved sequentially until the solution convergence is achieved. For the up-winding strategy, first order up-winding is applied for the turbulent kinetic energy and dissipation rate, and second order up-winding is employed for the momentum.

Three meshes with different element numbers are employed for determining mesh-independent results. The coarse, medium, and dense meshes are composed of approximately 8,700, 26,000, and 88,400 triangular elements, respectively. For increasing the quality of the mesh, inflation layers are added on the wall proximity for increasing the solution accuracy. For a control heart at week 23, the area weighted average vorticity levels in the left heart during one full cardiac cycle are determined as 43.43, 47.92, and 48.47 s^−1^ for the coarse, medium, and dense meshes, respectively. Since the difference between the average vorticity levels of medium and dense meshes are lower than 2%, the results of the medium mesh are accepted to be satisfactorily accurate (Kelsey et al., 2017). For further CFD simulations, the medium mesh with approximately 26,000 elements is used to determine the flow field in the heart.

## Results

In this section, the findings of control and HLHS hearts are compared and analyzed using three distinct methods described in the preceding section.

### Comparison of Chamber Areas for Healthy and Hypoplastic Left Heart Syndrome Hearts

**FIGURE 2:**
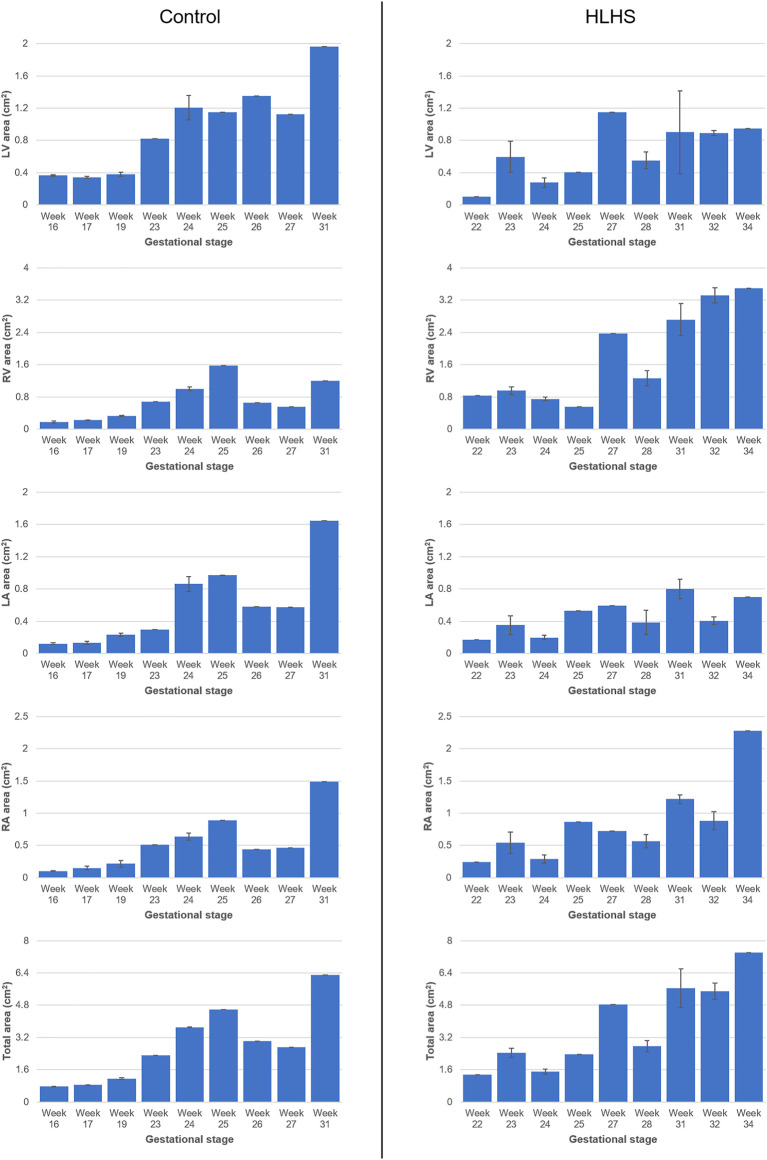
Cross-sectional areas of the heart chambers for the control and HLHS hearts at various gestational stages.

The cross-sectional area of each heart chamber is determined at the instant of the left ventricular diastolic phase and provided in Tables 2 and 3 for the control and HLHS hearts, respectively. In Figure 2, the total cross-sectional areas of the control and HLHS hearts are represented at different gestational stages.

**TABLE 2:** Cross-sectional areas of the heart chambers for control hearts at various gestational stages.

Gestational stage	LV area (cm^2^)	RV area (cm^2^)	LA area (cm^2^)	RA area (cm^2^)	Total area (cm^2^)
Week 16	0.352	0.216	0.108	0.108	0.784
Week 16	0.381	0.148	0.133	0.099	0.761
Week 17	0.357	0.228	0.155	0.111	0.850
Week 17	0.322	0.231	0.108	0.190	0.850
Week 19	0.413	0.347	0.209	0.146	1.115
Week 19	0.344	0.315	0.260	0.286	1.205
Week 23	0.821	0.677	0.297	0.513	2.308
Week 24	0.887	1.068	1.085	0.672	3.712
Week 24	1.525	0.893	0.734	0.511	3.663
Week 24	1.208	1.048	0.766	0.737	3.758
Week 25	1.151	1.580	0.970	0.893	4.594
Week 26	1.353	0.659	0.582	0.436	3.030
Week 27	1.121	0.554	0.573	0.463	2.711
Week 31	1.963	1.203	1.643	1.489	6.299

**TABLE 3:** Cross-sectional areas of the heart chambers for HLHS hearts at various gestational stages.

Gestational stage	LV area (cm^2^)	RV area (cm^2^)	LA area (cm^2^)	RA area (cm^2^)	Total area (cm^2^)
Week 22	0.102	0.836	0.168	0.241	1.347
Week 23	0.868	1.083	0.518	0.305	2.774
Week 23	0.326	0.825	0.184	0.776	2.111
Week 24	0.422	0.788	0.216	0.389	1.815
Week 24	0.187	0.815	0.131	0.135	1.268
Week 24	0.218	0.638	0.244	0.345	1.446
Week 25	0.404	0.559	0.527	0.865	2.356
Week 27	1.147	2.376	0.593	0.727	4.844
Week 28	0.566	1.726	0.129	0.376	2.796
Week 28	0.322	1.057	0.274	0.526	2.179
Week 28	0.771	1.005	0.745	0.796	3.317
Week 31	1.630	3.277	0.972	1.124	7.003
Week 31	0.172	2.154	0.634	1.316	4.275
Week 32	0.846	3.043	0.342	0.695	4.926
Week 32	0.937	3.594	0.470	1.076	6.076
Week 34	0.950	3.495	0.698	2.275	7.417

**FIGURE 3:**
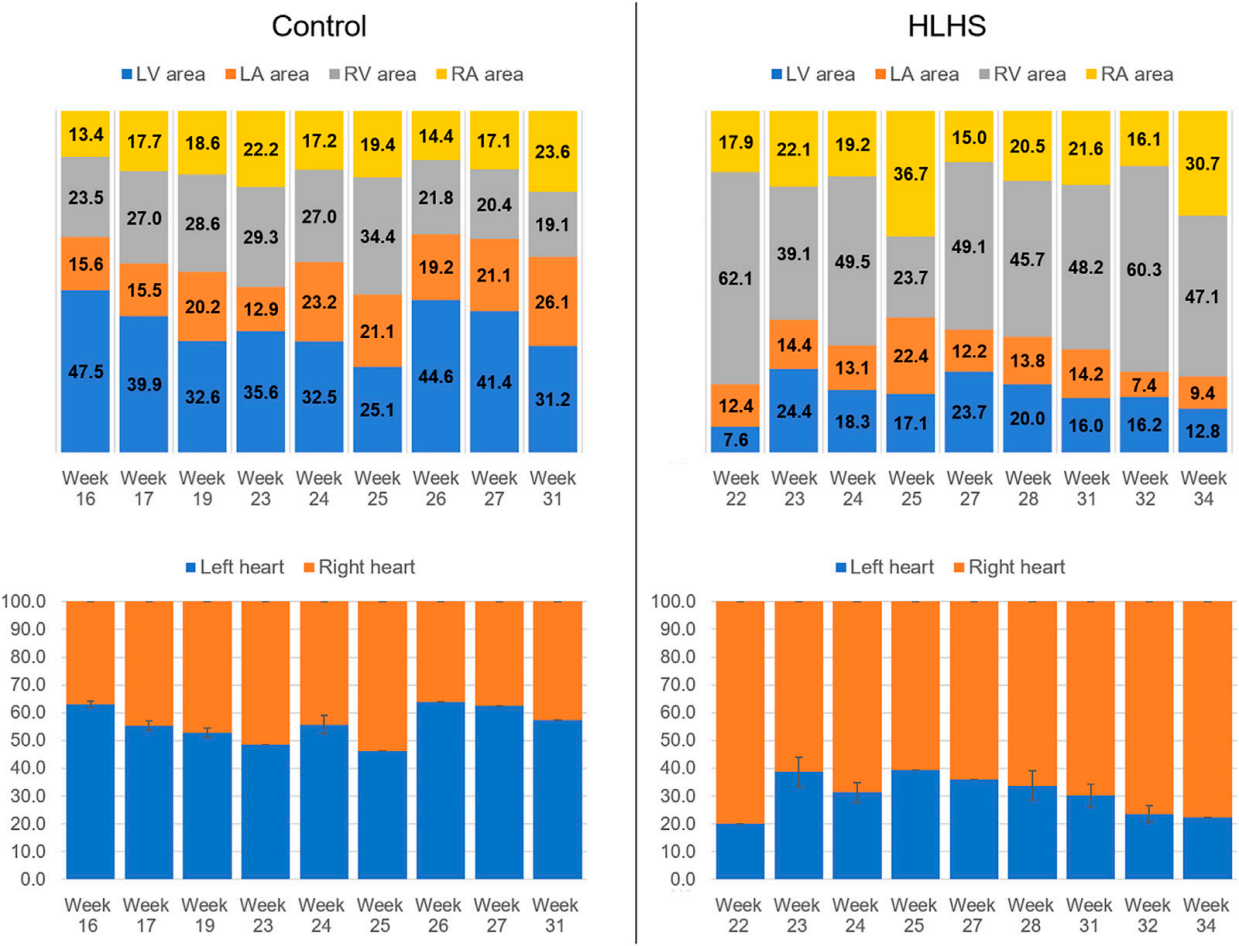
Percent chamber areas and the area ratios between the left and right sides of the control and HLHS hearts.

The percent cross-sectional area of each heart chamber is determined by dividing the chamber area to the total cross-sectional area of the heart. Figure 3 shows the percent chamber area and the ratio between the left and right sides of the heart for the control and HLHS hearts. The area of the right side is obtained by summing the cross-sectional areas of RV and RA. Similarly, the area of the left side is calculated by adding the cross-sectional areas of LV and LA.

**FIGURE 4:**
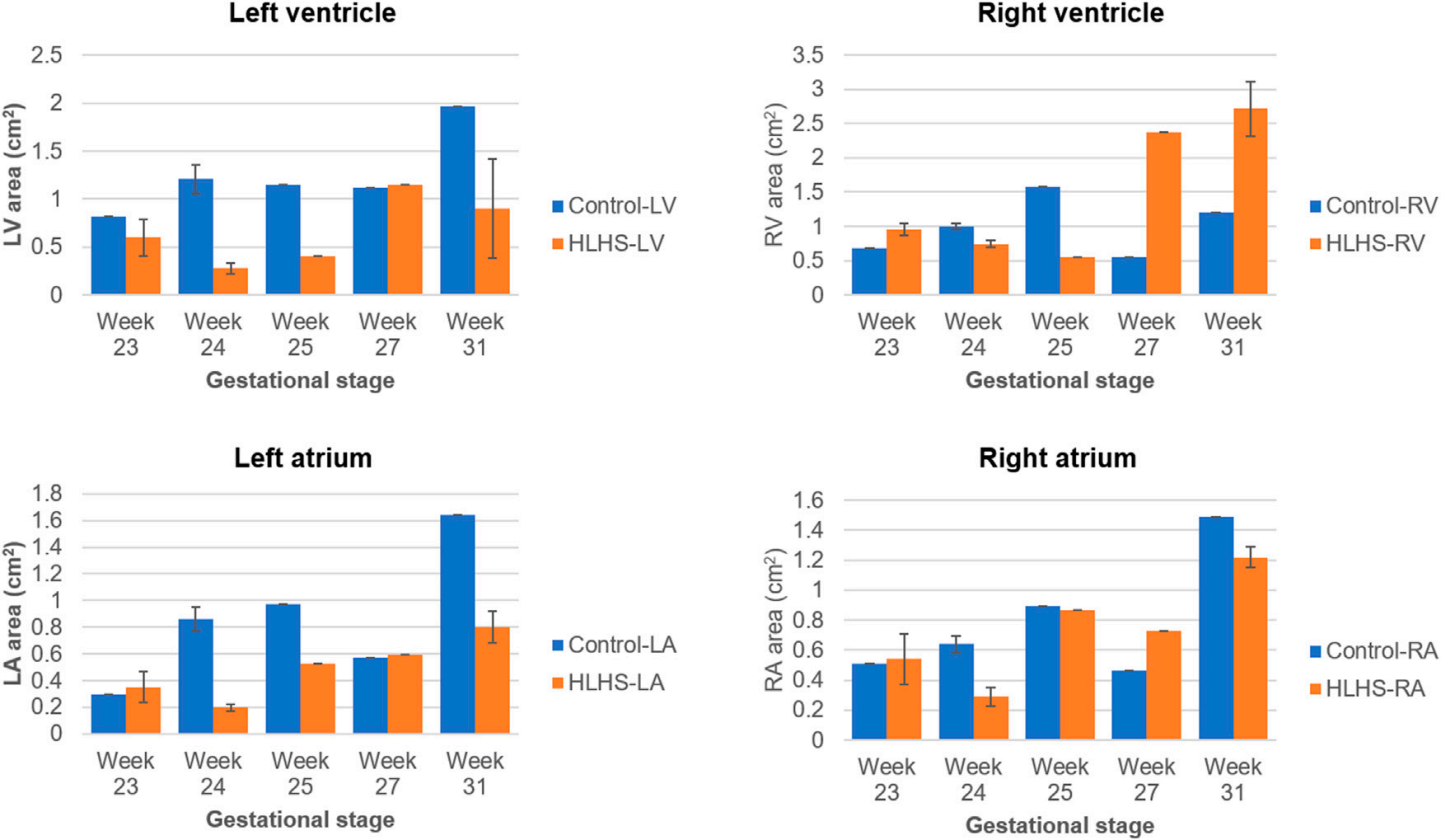
Comparison of cross-sectional areas between the control and HLHS hearts considering various gestational stages.

The common gestational stages within the investigated control and HLHS hearts are the weeks 23, 24, 25, 27, and 31. Considering these five different gestational weeks, the average cross-sectional areas of the chambers are compared between the control and HLHS hearts as given in Table 4. The statistical analyses are performed using two tailed Student’s *t* test. The *p* values less than 0.05 are considered to be statistically significant. Considering the cross-sectional chamber areas within week 23 and week 31, the *p* values are determined as 0.0485 for LV, 0.431 for RV, 0.105 for LA, 0.553 for RA, and 0.525 for the total cross-sectional area of the heart. A significant difference is only observed for the cross-sectional area of LV between the control and HLHS hearts. The findings presented in Table 4 are also depicted in Figure 4 for a clearer comparison of control and HLHS cases.

**TABLE 4:** The comparison of averaged heart chamber cross-sectional areas between the control and HLHS hearts. The values given in parenthesis are the percentages with respect to the total cross-sectional area of the fetal heart.

Cross-sectional area (cm^2^)	Week 23	Week 24	Week 25	Week 27	Week 31
Control-LV	0.821 (35.6%)	1.207 (32.5%)	1.151 (25.1%)	1.121 (41.4%)	1.963 (31.2%)
Control-RV	0.677 (29.3%)	1.003 (27.0%)	1.580 (34.4%)	0.554 (20.4%)	1.203 (19.1%)
Control-LA	0.297 (12.9%)	0.861 (23.2%)	0.970 (21.1%)	0.573 (21.1%)	1.643 (26.1%)
Control-RA	0.513 (22.2%)	0.640 (17.2%)	0.893 (19.4%)	0.463 (17.1%)	1.489 (23.6%)
Control-total	2.308 (100%)	3.711 (100%)	4.594 (100%)	2.711 (100%)	6.299 (100%)
HLHS-LV	0.597 (24.4%)	0.276 (18.3%)	0.404 (17.1%)	1.147 (23.7%)	0.901 (16.0%)
HLHS-RV	0.954 (39.1%)	0.747 (49.5%)	0.559 (23.7%)	2.376 (49.1%)	2.715 (48.2%)
HLHS-LA	0.351 (14.4%)	0.197 (13.1%)	0.527 (22.4%)	0.593 (12.2%)	0.803 (14.2%)
HLHS-RA	0.540 (22.1%)	0.290 (19.2%)	0.865 (36.7%)	0.727 (15.0%)	1.220 (21.6%)
HLHS-total	2.442 (100%)	1.510 (100%)	2.356 (100%)	4.844 (100%)	5.639 (100%)

### Comparison of Flow Waveforms at Mitral and Tricuspid Valves

**FIGURE 5:**
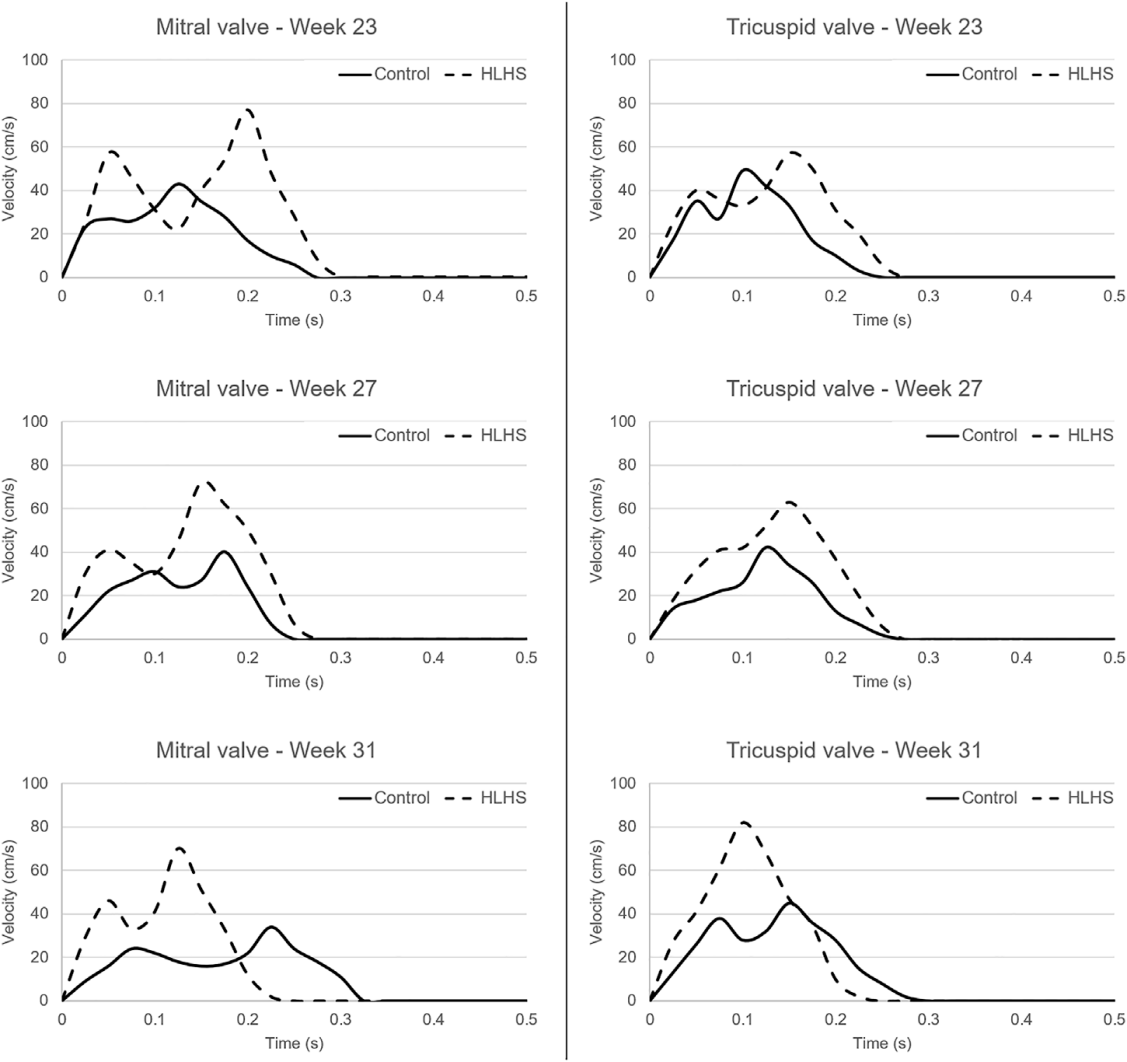
Flow waveforms in the mitral and tricuspid valves of the control and HLHS hearts at gestational weeks 23, 27, and 31.

**FIGURE 6:**
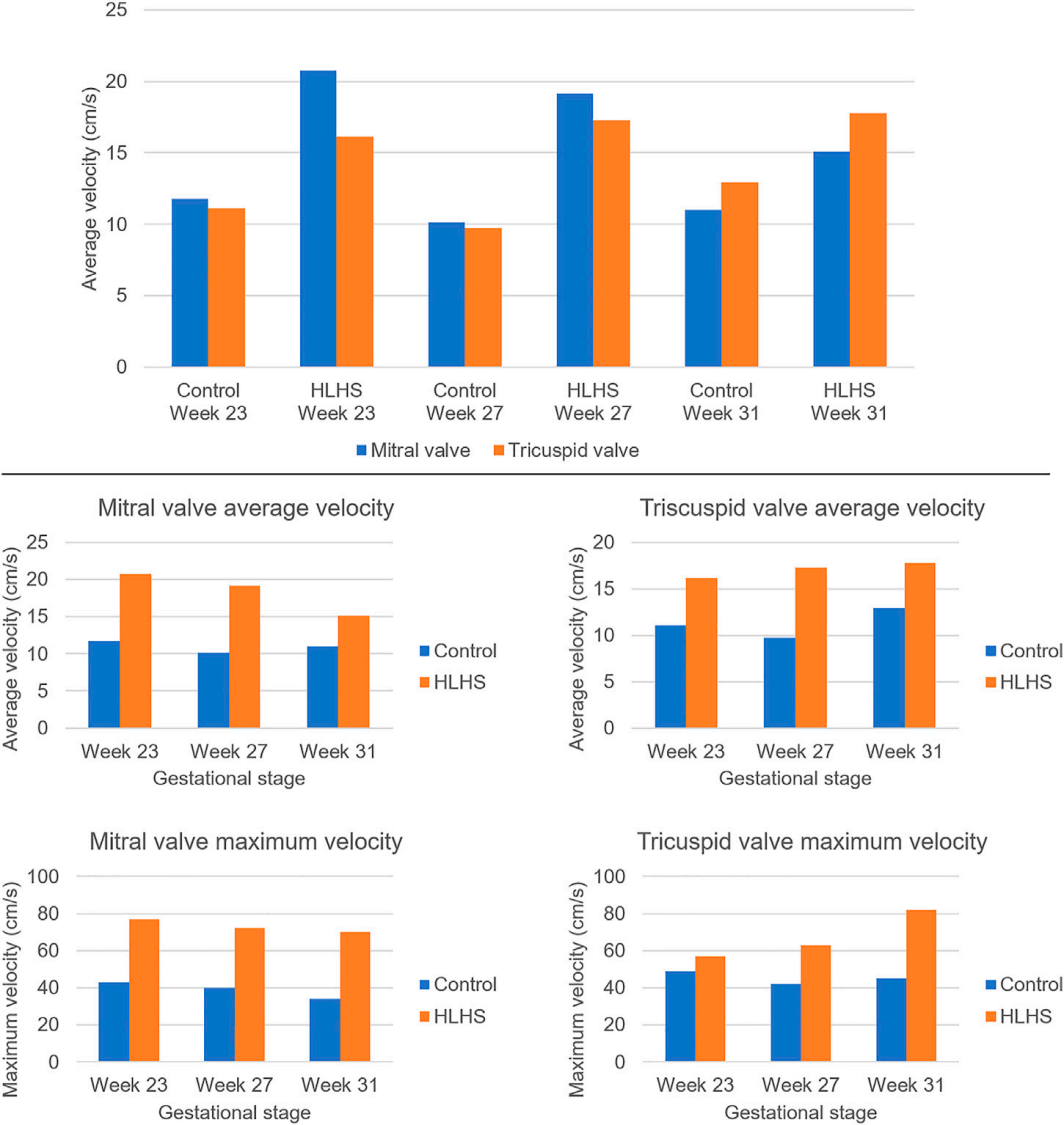
Temporally averaged velocities in the mitral and tricuspid valves of the control and HLHS hearts at gestational stages of week 23, week 27, and week 31. The temporal averages are determined using the velocity levels within one full cardiac cycle.

The atrioventricular valve flow waveforms are compared between the control and HLHS hearts at weeks 23, 27, and 31. The velocity waveforms at these gestational weeks are presented in Figure 5 for the mitral and tricuspid valves. The mitral valve serves as a bridge between LA and LV in the left heart. The tricuspid valve connects RA and RV in the right heart. The velocity profiles show an important difference between the control and HLHS hearts. In the mitral and tricuspid valves of the control hearts, the peak velocity is observed around 40 cm/s for weeks 23, 27, and 31, and the alteration is not significant between different gestational stages. In case of HLHS, the peak velocity in mitral valve is obtained within 70–80 cm/s, indicating a severe increase in the velocity levels. Similarly, the peak velocity levels in the tricuspid valve are also increased for HLHS hearts, where the peak velocities are determined within the range of 60–80 cm/s. Valve flow velocities approach to nearly zero after 0.3 s in the cardiac cycle, and the peak velocities are generally determined between 0.1 and 0.2 s. The temporally averaged velocity of each flow waveform is calculated using the values within one full cardiac cycle. The temporal average velocities are presented in Figure 6 for the mitral and tricuspid valves of the control and HLHS hearts. The relative increase in the average and maximum valvular velocities of HLHS hearts compared to that of the controls can be better observed in Figure 6.

The average velocities within the cardiac cycle are determined around 12 cm/s and 11 cm/s in the mitral and tricuspid valves of a 23 week control heart, respectively. These average velocities are determined around 10 cm/s in both the mitral and tricuspid valves of a 27 week control heart. For a 31 week control heart, the average velocities are around 11 cm/s in the mitral valve and 13 cm/s in the tricuspid valve, which is showing that there is no significant change in the average valve flow velocities of control hearts throughout the developmental stages. On the other hand, the average velocities in the mitral valve of HLHS hearts are determined around 21 cm/s at week 23, 19 cm/s at week 27, and 15 cm/s at week 31, indicating a systematic reduction depending on the developmental stage. The average tricuspid velocities of HLHS hearts demonstrate an increase with the gestational development, where the average velocities are determined as 16 cm/s at week 23, 17.5 cm/s at week 27, and 18 cm/s at week 31.

The valve flow profiles presented in Figure 5 are averaged waveforms and the highest activity is observed within the first 0.3 s of the cardiac cycles. The velocities within the range of 0–0.3 s are statistically compared for the control and HLHS hearts. For the mitral valve, the *p*-values are determined as 0.067, 0.069, and 0.351 at week 23, week 27, and week 31, respectively. This indicates that the differences between the control and HLHS mitral valves are not statistically different for the investigated weeks. However, the comparison of tricuspid valve flow profiles within 0–0.3 s resulted in *p*-values of 0.022, 0.005, and 0.049 at week 23, week 27, and week 31, which reveals that the changes in tricuspid flow rates are statistically different between the control and HLHS fetal hearts.

### Comparison of Hemodynamics Using Computational Fluid Dynamics Simulations

**FIGURE 7:**
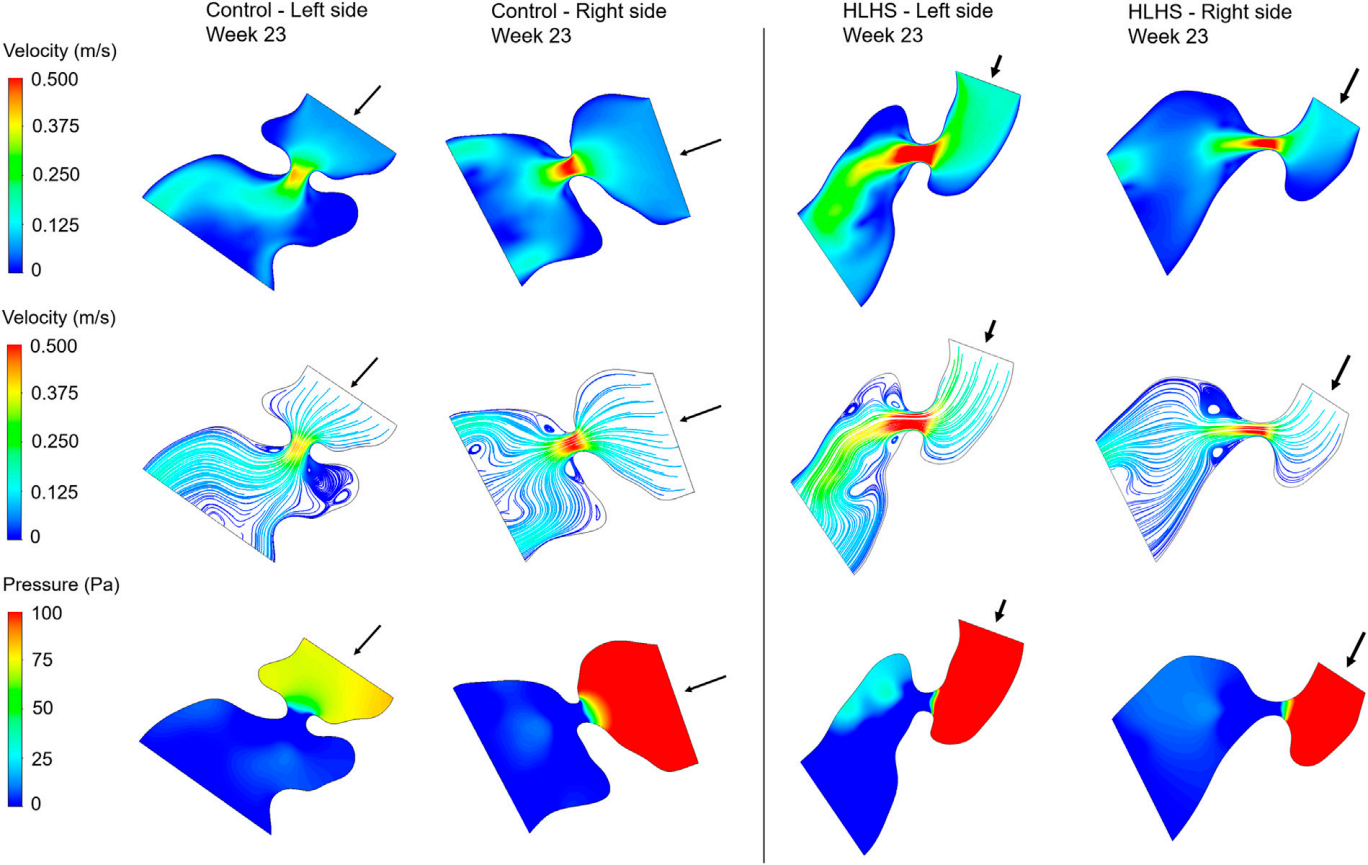
Velocity contours, flow streamlines, and pressure contours at the instant of peak inlet flow rate for 23-week control and HLHS hearts. The arrows indicate the flow direction at the inlet.

**FIGURE 8:**
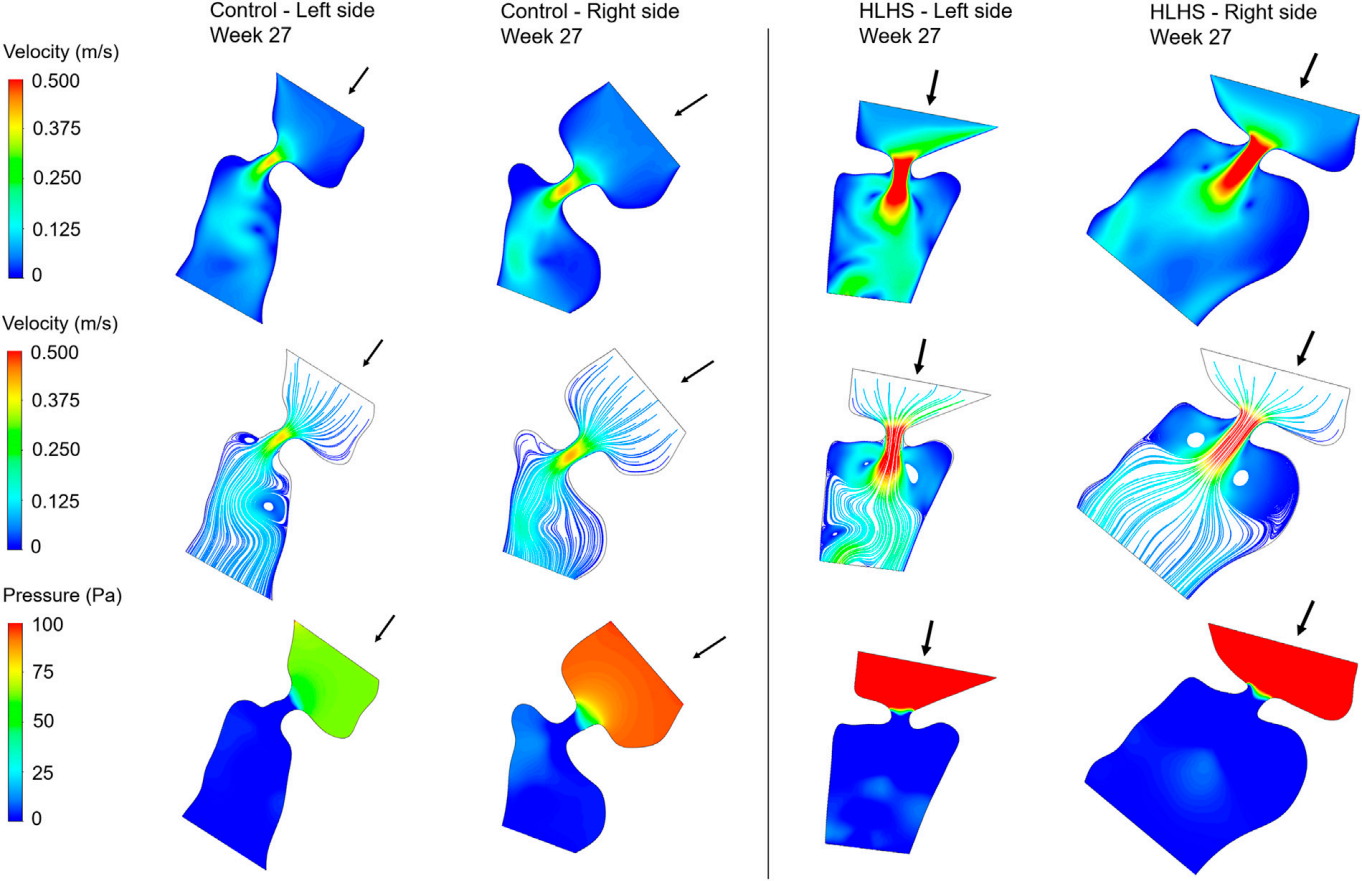
Velocity contours, flow streamlines, and pressure contours at the instant of peak inlet flow rate for 27-week control and HLHS hearts. The arrows indicate the flow direction at the inlet.

**FIGURE 9:**
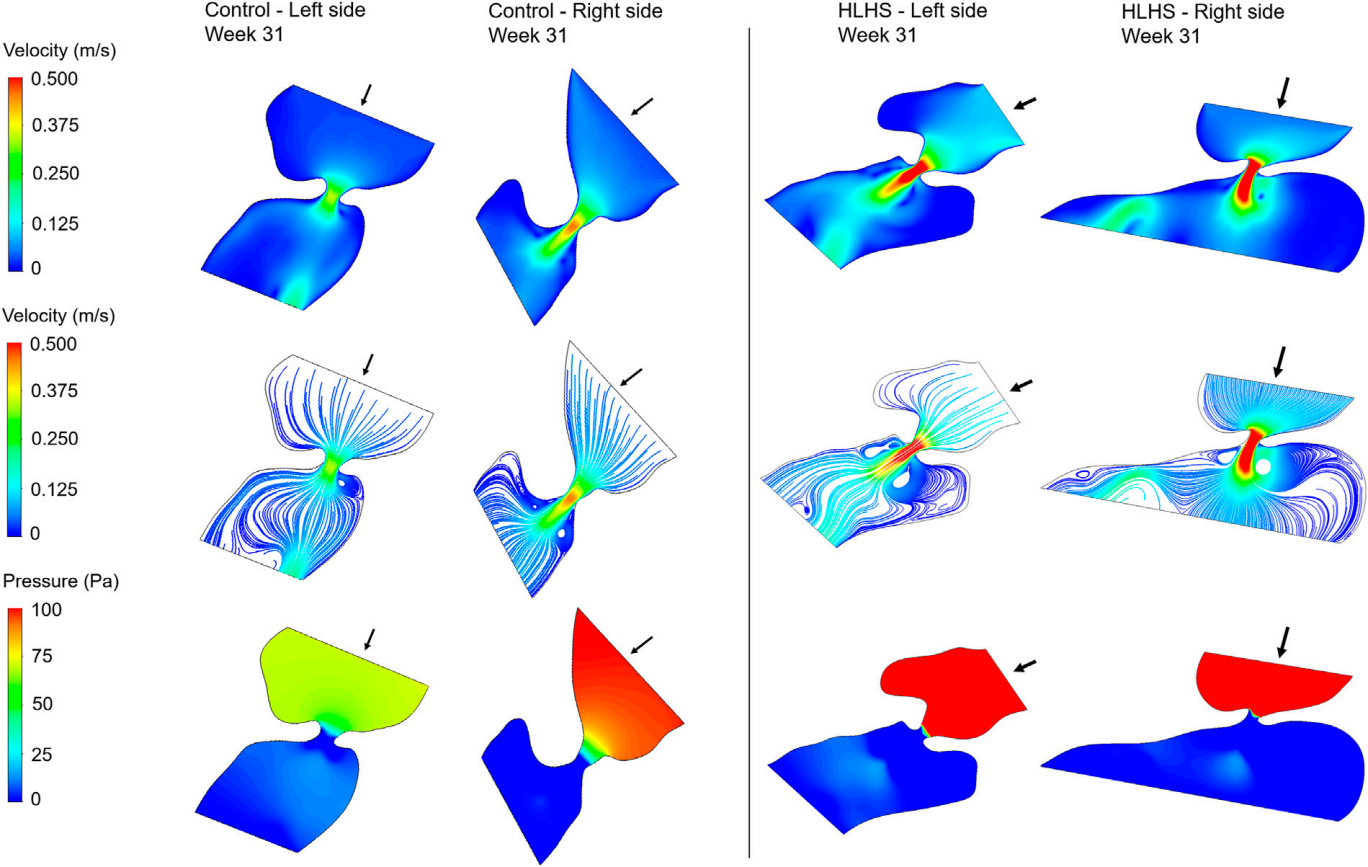
Velocity contours, flow streamlines, and pressure contours at the instant of peak inlet flow rate for 31-week control and HLHS hearts. The arrows indicate the flow direction at the inlet.

Hemodynamic quantifications are performed to compare the flow conditions in healthy and HLHS hearts. Similar to the previous comparisons, the control and HLHS hearts at weeks 23, 27, and 31 are used for the hemodynamic analysis. Velocity magnitudes, flow streamlines, pressures, and WSS levels are investigated to determine the HLHS-related disturbances. In Figures 7–9, the velocity and pressure contours are presented at the instant of peak inlet flow rate for 23-week, 27-week, and 31-week control and HLHS fetal hearts, respectively. The instant of peak inlet flow is the most critical moment, due to generating the highest velocity magnitudes and WSS levels in the flow domain. In addition, the CFD findings are expected to be more reliable at the instant of peak flow conditions with a fully open valve configuration due to the static walls of the CFD models which do not change their shapes depending on time.

Flow velocities significantly increase at the proximity of the valves, which results in high velocity gradients. This sudden change in velocities around the valves leads to a high amount of pressure drop. In both sides of the 23-week, 27-week, and 31-week hearts, the peak valve flow velocities are observed to be higher in HLHS hearts. Due to the higher peak velocities, the pressure drop across the valves are higher for the HLHS hearts. For the analyzed control hearts, the mitral valve in the left heart experiences a lower pressure drop compared to the tricuspid valve in the right heart, mainly due to the lower peak velocities. The magnitude of pressure drop is similar in the mitral and tricuspid valves of investigated HLHS hearts.

**FIGURE 10:**
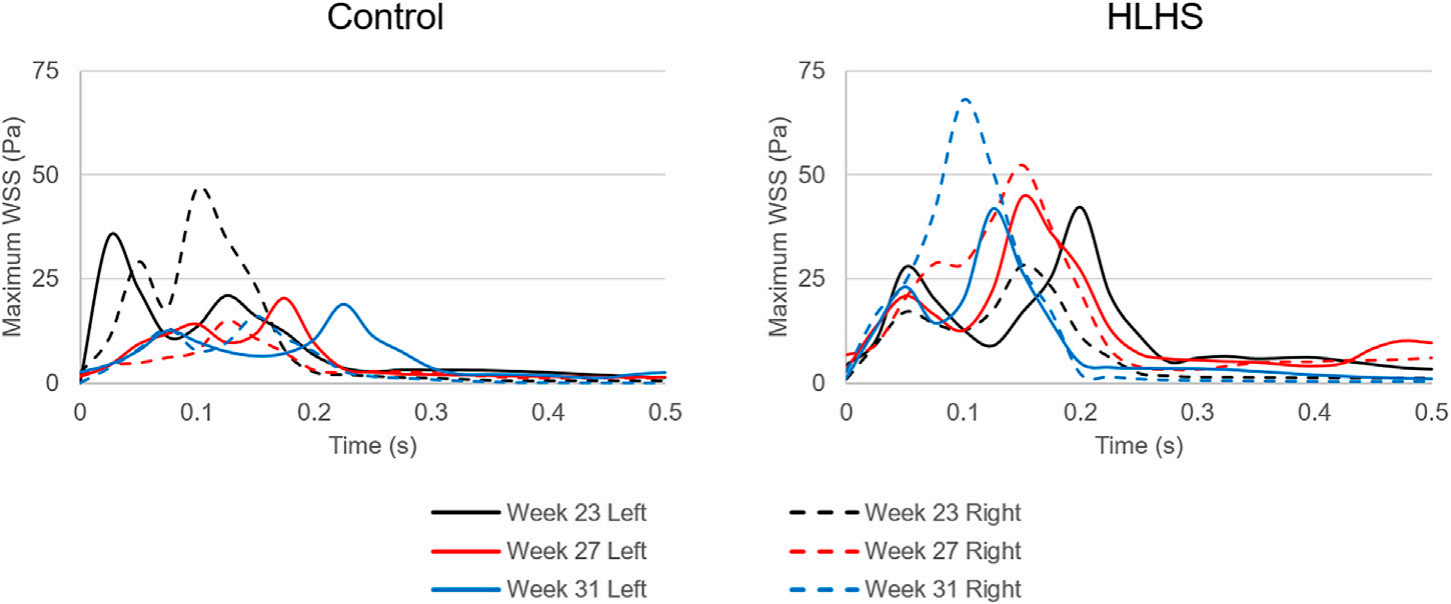
Maximum WSS levels for the left and right sides of the control and HLHS hearts at weeks 23, 27, and 31.

**FIGURE 11:**
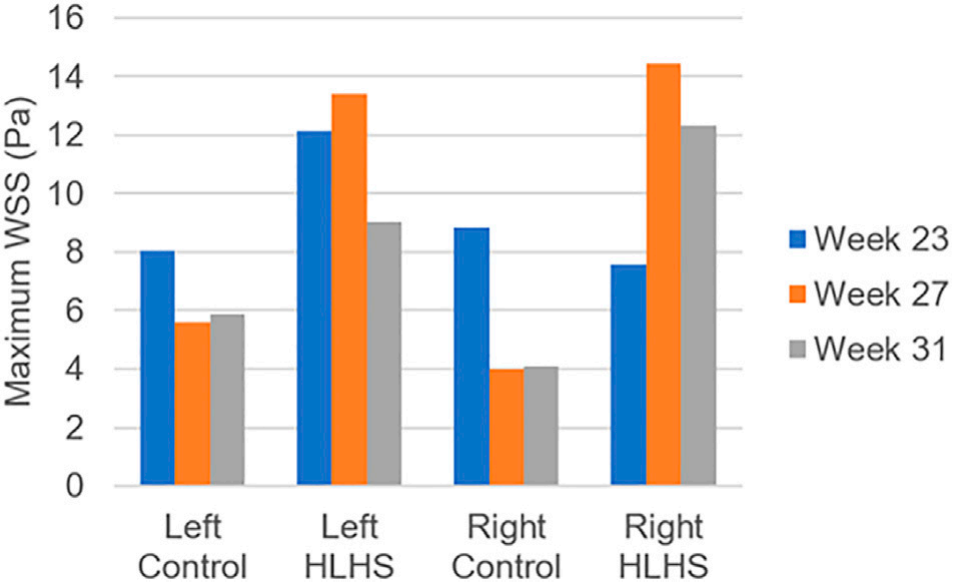
Temporal averages of maximum WSS levels during one cardiac cycle for the control and HLHS hearts at weeks 23, 27, and 31.

In Figure 10, the maximum WSS levels of control and HLHS hearts are presented as a function of time considering one full cardiac cycle. In the heart models, the maximum WSS is always expected around the valves due to the reduced flow area at the valve proximity. The highest activities are seen in the first 0.3 s of the cardiac cycle. It is observed that the maximum WSS levels tend to decrease after 0.3 s in both the control and HLHS hearts. The highest WSS levels are determined at week 23 when compared to weeks 27 and 31. When the maximum WSS levels of HLHS hearts are investigated, it is observed that a general increase in WSS levels is observed for the HLHS hearts compared to those of the controls. The time-dependent profiles of maximum WSS show a close resemblance with the valve flow profiles, since WSS is directly related to the friction component of the flow velocity. In order to elucidate the HLHS-related differences more clearly, the results presented in Figure 10 are used to determine the temporal averages within one cardiac cycle. Figure 11 shows the temporal averages of maximum WSS levels, as well as the ratios between the left and right sides of fetal hearts. The ratios of maximum WSS between the two sides of the hearts are determined by averaging the findings at weeks 23, 27, and 31. For the left side of the control hearts, the temporal average of maximum WSS is determined between 6 and 8 Pa. This maximum WSS range is observed to be expanded for the right side of control hearts with a range of 4–9 Pa. For the HLHS hearts, the temporal averages of maximum WSS are determined within 9–13.5 Pa for the left side and within 7.6–14.2 Pa for the right side. In Figure 11, the maximum WSS findings are represented for a clearer comparison between the left and right sides of the control and HLHS hearts at different gestational stages.

The maximum WSS ratio in Figure 11 is determined by averaging the maximum WSS levels at weeks 23, 27, and 31. The left to right ratio of maximum WSS in the control hearts are determined as 47.6–52.4%, 58.4–41.6%, and 59.0–41.0% at weeks 23, 27, and 31, respectively. This left to right ratio is found as 61.6–38.4%, 48.1–51.9%, and 42.2–57.8% for the HLHS hearts at week 23, 27, and 31, which is indicating the increasing WSS load on the right side of HLHS hearts with the fetal development.

## Discussion

In this study, the fetal development of healthy and HLHS hearts are investigated by comparing the cross-sectional areas of the heart chambers, measuring the flow profiles in mitral and tricuspid valves, and analyzing the hemodynamic parameters using CFD simulations. Various gestational stages between week 16 and week 34 are considered in the investigations. The comparison of healthy and HLHS hearts reveals important differences in terms of hemodynamics.

The heart chamber dimensions systematically increase with the fetal development of control hearts; however, the formation of HLHS inhibits the growth of LA and LV. The ratio of the left side cross-sectional area to the entire cross-sectional area of the heart is determined within 46–63% for the controls during the gestational stages between weeks 16 and 31. This cross-sectional area ratio between the left side and the entire heart remains between 20 and 40% for HLHS hearts during the weeks 22–34, indicating a significant underdevelopment in the left heart.

The echocardiography measurements in the heart valves show that HLHS increases the peak flow rates both in the mitral and tricuspid valves. For the HLHS heart at week 23, the average velocity in the mitral valve is higher than the tricuspid valve, where the average velocity during one cardiac cycle is measured around 21 cm/s in the mitral valve and 16 cm/s in the tricuspid valve. At week 27, the average valve velocities in HLHS hearts are determined as 19 cm/s in the mitral valve and 17 cm/s in the tricuspid valve, indicating that the excess flow in the mitral valve is decreasing with the gestational development. At week 31 of HLHS heart, the average velocity is approximately 15 cm/s in the mitral valve and 18 cm/s in the tricuspid valve. This fact shows that, in case of HLHS, the average flow velocity decreases in the mitral valve and increases in the tricuspid valve as the heart develops. The comparison of valve flow rates within 0–0.3 s reveals that the changes in the mitral valve are not significant between the control and HLHS hearts. Nevertheless, the flow profile differences in the tricuspid valve are statistically significant, which explains the need of tricuspid valve repair in twenty-five percent of HLHS patients (Mah et al., 2021).

The hemodynamic analyses also confirm the altered biomechanical environment in HLHS hearts compared to the controls. The pressure drop across the heart valves are higher in HLHS hearts due to the higher valve flow velocities. The maximum WSS levels are investigated around the valve proximity and an increased maximum WSS is observed for the HLHS fetal hearts compared to the controls. For the left and right sides of healthy hearts, the maximum WSS level at week 23 is greater than the maximum WSS levels at weeks 27 and 31. For HLHS hearts, the maximum WSS level tends to decrease at the left side as the heart develops. Opposingly, the maximum WSS level at the right side of HLHS hearts tends to increase with gestational development. When the maximum WSS levels are compared by averaging the results at weeks 23, 27, and 31, it is observed that there is a balance between the left and right sides both in the control and HLHS hearts. However, with the development of HLHS hearts, an increasing maximum WSS load in the right side begins to deteriorate the balance between the left and right sides. The level of WSS is an important parameter for the developmental dynamics of the heart, since WSS levels are sensed by the endothelial cells (Lee et al., 2018). This way, the hemodynamic disturbances and mechanobiological forces play a role in the formation of CHDs in a side-specific manner.

In a previous study, we investigated the embryonic chicken hearts with left atrial ligation (LAL) which resembled HLHS in human fetal hearts, and we determined that the maximum WSS balance was deteriorated with a higher WSS load in the right side of LAL chicken hearts (Salman et al., 2021a). For the chicken embryo hearts, the left to right ratio of maximum WSS levels were determined as 21.7–78.3% at day-7 of incubation. In the current study, similarly, the right side of HLHS heart experiences a higher maximum WSS compared to the left side as the fetus develops, but with a limited increase compared to the deterioration in the embryonic chicken hearts with LAL. The left to right ratio of maximum WSS in 31-week HLHS hearts are determined as 42.2–57.8%. The reason of this difference between the two studies can be explained by the investigated period of the embryos. In the previously investigated LAL chicken hearts (Salman et al., 2021a), we elucidated the period up to 7-days of incubation, which approximately corresponds to the first third of the 21-day total incubation period. In the current CFD models, we investigated human HLHS fetal hearts between the weeks 23 and 31, which is corresponding to the second half of the pregnancy. This may be due to the fact that the difference between maximum WSS levels at the right and left sides of the heart is compensated with the morphological changes in the later stages of the development.

There are several limitations in this work. Due to the limited number of data, up to three hearts are examined at each interested gestational week. The lack of data prevented to perform a comprehensive statistical analysis. In order to capture the differences between the healthy and HLHS hearts in a systematic way, the samples are selected at the gestational weeks of 23, 27, and 31 with 4-week increments between each other. The addition of more sample hearts in future studies is important to confirm the accuracy of the findings.

The determination of chamber geometries is performed manually using 2D ultrasound images at the ventricular diastolic phase. Detecting the exact heart chamber borders is challenging for some blurry fetal images which can introduce an error in the boundaries of the CFD models. Expanding the number of sample embryonic hearts would also be beneficial for minimizing the possible errors of the CFD simulations.

The employed CFD models have static boundaries with no displacement and flexibility, which neglects the deformation of the heart structures. Therefore, the CFD results are expected to be accurate only during the instant of the left ventricular filling phase, particularly around the valve proximity. As a consequence, our findings mainly concentrate at the instant of peak valve flow rate and maximum WSS around the valves. The determination of chamber and valve borders are performed using the multiple ultrasound images and movies. In further images, an image-processing based tool is aimed to be developed for reducing the dimensional errors in the CFD model geometries. The use of 2D geometries is another limitation in CFD models, because 3D models can better predict the chaotic flow in the chambers. Wiputra et al. (2018) employed 3D CFD modeling with moving chamber boundaries and reported a time-averaged WSS within 0.51–0.65 Pa in LV of 31-week healthy fetuses during the diastolic phase. In our simulations, the average WSS in LV of a 31-week control fetal heart is determined as 0.48 Pa, which is showing that the error is acceptable through the left ventricular diastolic phase. The use of non-Newtonian blood models with shear-dependent variable viscosity can also provide better prediction of hemodynamics in further investigations. Nevertheless, the findings provide an insight about the fetal cardiac development and reveal that the biomechanical environment is significantly altered in case of HLHS formation.

## Conclusion

In this work, we performed comparisons between healthy and HLHS human fetal hearts in order to reveal the differences during the gestational development stages in terms of cross-sectional heart chamber dimensions, mitral and tricuspid valve flow rates, and hemodynamics. The comparisons between the healthy and HLHS hearts are mainly carried out at weeks 23, 27, and 31. It is seen that there is a balance between the cross-sectional areas of the left and right sides of healthy hearts. On the other hand, an unbalance in the cross-sectional area is observed between the two sides of HLHS hearts, which indicates an underdeveloped left heart. Due to this unbalance in the HLHS hearts, hemodynamic parameters such as flow velocity, pressure, and WSS levels are altered and the biomechanical environment is significantly changed. For the HLHS hearts, the maximum WSS levels tend to decrease in the left side and increase in the right side with the gestational development, which indicates a difference between the WSS environments of healthy and HLHS hearts.

## Data Availability

The original contributions presented in the study are included in the article/Supplementary Material, further inquiries can be directed to the corresponding author.

## References

[B1] Barron D. J. Kilby M. D. Davies B. Wright J. G. Jones T. J. Brawn W. J. (2009). Hypoplastic Left Heart Syndrome. The Lancet 374 (9689), 551–564. 10.1016/S0140-6736(09)60563-8 19683641

[B2] Bäumler K. Vedula V. Sailer A. M. Seo J. Chiu P. Mistelbauer G. (2020). Fluid-Structure Interaction Simulations of Patient-Specific Aortic Dissection. Biomech. Model. Mechanobiol 19 (5), 1607–1628. 10.1007/s10237-020-01294-8 31993829

[B3] Benslimane F. M. Alser M. Zakaria Z. Z. Sharma A. Abdelrahman H. A. Yalcin H. C. (2019). Adaptation of a Mice Doppler Echocardiography Platform to Measure Cardiac Flow Velocities for Embryonic Chicken and Adult Zebrafish. Front. Bioeng. Biotechnol. 7, 96. 10.3389/fbioe.2019.00096 31139625 PMC6527763

[B4] Bove E. L. de Leval M. R. Migliavacca F. Guadagni G. Dubini G. (2003). Computational Fluid Dynamics in the Evaluation of Hemodynamic Performance of Cavopulmonary Connections after the norwood Procedure for Hypoplastic Left Heart Syndrome. J. Thorac. Cardiovasc. Surg. 126 (4), 1040–1047. 10.1016/S0022-5223(03)00698-6 14566244

[B5] Chen Z. Zhou Y. Wang J. Liu X. Ge S. He Y. (2017). Modeling of Coarctation of Aorta in Human Fetuses Using 3D/4D Fetal Echocardiography and Computational Fluid Dynamics. Echocardiography 34 (12), 1858–1866. 10.1111/echo.13644 28833523

[B6] Courchaine K. Gray M. Beel K. Thornburg K. Rugonyi S. (2019). 4-D Computational Modeling of Cardiac Outflow Tract Hemodynamics Over Looping Developmental Stages in Chicken Embryos. J. Cardiovasc. Develop. Dis. 6 (1), 11. 10.3390/jcdd6010011 PMC6463052 30818869

[B7] Courchaine K. Rugonyi S. (2018). Quantifying Blood Flow Dynamics during Cardiac Development: Demystifying Computational Methods. Phil. Trans. R. Soc. B 373 (1759), 20170330. 10.1098/rstb.2017.0330 30249779 PMC6158206

[B8] Courchaine K. Rykiel G. Rugonyi S. (2018). Influence of Blood Flow on Cardiac Development. Prog. Biophys. Mol. Biol. 137, 95–110. 10.1016/j.pbiomolbio.2018.05.005 29772208 PMC6109420

[B9] DeGroff C. G. Thornburg B. L. Pentecost J. O. Thornburg K. L. Gharib M. Sahn D. J. (2003). Flow in the Early Embryonic Human Heart. Pediatr. Cardiol. 24 (4), 375–380. 10.1007/s00246-002-0343-9 12632224

[B10] Dempster N. Cua C. L. Wernovsky G. Caris E. Neely T. Allen R. (2017). Children with Hypoplastic Left Heart Syndrome Have Lower Quality of Life Than Healthy Controls and Children with Other Illnesses. Cardiol. Young 28 (1), 21–26. 10.1017/S1047951117001159 28847316

[B11] Dyer L. A. Rugonyi S. (2021). Fetal Blood Flow and Genetic Mutations in Conotruncal Congenital Heart Disease. J. Cardiovasc. Develop. Dis. 8 (8), 90. 10.3390/jcdd8080090 PMC8397097 34436232

[B12] Hoffman J. I. E. Kaplan S. (2002). The Incidence of Congenital Heart Disease. J. Am. Coll. Cardiol. 39 (12), 1890–1900. 10.1016/S0735-1097(02)01886-7 12084585

[B13] Hoffman J. I. E. (2013). The Global Burden of Congenital Heart Disease : Review Article. Cardiovasc. J. Africa 24 (4), 141–145. 10.5830/cvja-2013-028 PMC3721933 24217047

[B14] Jarrell D. K. Lennon M. L. Jacot J. G. (2019). Epigenetics and Mechanobiology in Heart Development and Congenital Heart Disease. Diseases 7 (3), 52. 10.3390/diseases7030052 PMC6787645 31480510

[B15] Kelsey L. J. Powell J. T. Norman P. E. Miller K. Doyle B. J. (2017). A Comparison of Hemodynamic Metrics and Intraluminal Thrombus Burden in a Common Iliac Artery Aneurysm. Int. J. Numer. Meth. Biomed. Engng. 33 (5), e2821. 10.1002/cnm.2821 27509188

[B16] Lai C. Q. Lim G. L. Jamil M. Mattar C. N. Z. Biswas A. Yap C. H. (2016). Fluid Mechanics of Blood Flow in Human Fetal Left Ventricles Based on Patient-Specific 4D Ultrasound Scans. Biomech. Model. Mechanobiol 15 (5), 1159–1172. 10.1007/s10237-015-0750-5 26676944

[B17] Lee J. Vedula V. Baek K. I. Chen J. Hsu J. J. Ding Y. (2018). Spatial and Temporal Variations in Hemodynamic Forces Initiate Cardiac Trabeculation. JCI insight 3 (13), e96672. 10.1172/jci.insight.96672 PMC6124527 29997298

[B18] Mah K. Khoo N. S. Tham E. Yaskina M. Maruyama M. Martin B.-J. (2021). Tricuspid Regurgitation in Hypoplastic Left Heart Syndrome: Three-Dimensional Echocardiography Provides Additional Information in Describing Jet Location. J. Am. Soc. Echocardiography 34 (5), 529–536. 10.1016/j.echo.2020.12.010 33373699

[B19] Marsden A. L. Feinstein J. A. (2015). Computational Modeling and Engineering in Pediatric and Congenital Heart Disease. Curr. Opin. Pediatr. 27 (5), 587–596. 10.1097/MOP.0000000000000269 26262579 PMC4666705

[B20] Mäkikallio K. McElhinney D. B. Levine J. C. Marx G. R. Colan S. D. Marshall A. C. (2006). Fetal Aortic Valve Stenosis and the Evolution of Hypoplastic Left Heart Syndrome. Circulation 113 (11), 1401–1405. 10.1161/CIRCULATIONAHA.105.588194 16534003

[B21] Mutlu O. Salman H. E. Yalcin H. C. Olcay A. B. (2021). Fluid Flow Characteristics of Healthy and Calcified Aortic Valves Using Three-Dimensional Lagrangian Coherent Structures Analysis. Fluids 6 (6), 203. 10.3390/fluids6060203

[B22] Norwood W. I. (1991). Hypoplastic Left Heart Syndrome. Ann. Thorac. Surg. 52 (3), 688–695. 10.1016/0003-4975(91)90978-Y 1898174

[B23] Øyen N. Poulsen G. Boyd H. A. Wohlfahrt J. Jensen P. K. A. Melbye M. (2009). Recurrence of Congenital Heart Defects in Families. Circulation 120 (4), 295–301. 10.1161/CIRCULATIONAHA.109.857987 19597048

[B24] Pennati G. Socci L. Rigano S. Boito S. Ferrazzi E. (2008). Computational Patient-Specific Models Based on 3-D Ultrasound Data to Quantify Uterine Arterial Flow during Pregnancy. IEEE Trans. Med. Imaging 27 (12), 1715–1722. 10.1109/TMI.2008.924642 19033087

[B25] Roldán-Alzate A. García-Rodríguez S. Anagnostopoulos P. V. Srinivasan S. Wieben O. François C. J. (2015). Hemodynamic Study of TCPC Using *In Vivo* and *In Vitro* 4D Flow MRI and Numerical Simulation. J. Biomech. 48 (7), 1325–1330. 10.1016/j.jbiomech.2015.03.009 25841292 PMC4406283

[B26] Rugonyi S. (2016). Genetic and Flow Anomalies in Congenital Heart Disease. AIMS Genet. 03 (03), 157–166. 10.3934/genet.2016.3.157 PMC5417695 28480330

[B27] Rykiel G. López C. S. Riesterer J. L. Fries I. Deosthali S. Courchaine K. (2020). Multiscale Cardiac Imaging Spanning the Whole Heart and its Internal Cellular Architecture in a Small Animal Model. Elife, e58138. 33078706 10.7554/eLife.58138 PMC7595733

[B28] Salman H. E. Alser M. Shekhar A. Gould R. A. Benslimane F. M. Butcher J. T. (2021a). Effect of Left Atrial Ligation-Driven Altered Inflow Hemodynamics on Embryonic Heart Development: Clues for Prenatal Progression of Hypoplastic Left Heart Syndrome. Biomech. Model. Mechanobiol 20 (2), 733–750. 10.1007/s10237-020-01413-5 33481120 PMC7979615

[B29] Salman H. E. Kamal R. Y. Yalcin H. C. (2021b). Numerical Investigation of the Fetal Left Heart Hemodynamics during Gestational Stages. Front. Physiol. 12, 1488. 10.3389/fphys.2021.731428 PMC8458957 34566694

[B30] Salman H. E. Ramazanli B. Yavuz M. M. Yalcin H. C. (2019). Biomechanical Investigation of Disturbed Hemodynamics-Induced Tissue Degeneration in Abdominal Aortic Aneurysms Using Computational and Experimental Techniques. Front. Bioeng. Biotechnol. 7, 111. 10.3389/fbioe.2019.00111 31214581 PMC6555197

[B31] Salman H. E. Yalcin H. C. (2020). Advanced Blood Flow Assessment in Zebrafish via Experimental Digital Particle Image Velocimetry and Computational Fluid Dynamics Modeling. Micron 130, 102801. 10.1016/j.micron.2019.102801 31864139

[B32] Salman H. E. Yalcin H. C. (2021). Computational Modeling of Blood Flow Hemodynamics for Biomechanical Investigation of Cardiac Development and Disease. J. Cardiovasc. Develop. Dis. 8 (2), 14. 10.3390/jcdd8020014 PMC7912127 33572675

[B33] Saraf A. Book W. M. Nelson T. J. Xu C. (2019). Hypoplastic Left Heart Syndrome: From Bedside to Bench and Back. J. Mol. Cell Cardiol. 135, 109–118. 10.1016/j.yjmcc.2019.08.005 31419439 PMC10831616

[B34] Saw S. N. Dawn C. Biswas A. Mattar C. N. Z. Yap C. H. (2017). Characterization of the *In Vivo* wall Shear Stress Environment of Human Fetus Umbilical Arteries and Veins. Biomech. Model. Mechanobiol 16 (1), 197–211. 10.1007/s10237-016-0810-5 27456489

[B35] Wang L. W. Kesteven S. H. Huttner I. G. Feneley M. P. Fatkin D. (2018). High-Frequency Echocardiography-Transformative Clinical and Research Applications in Humans, Mice, and Zebrafish. Circ. J. 82 (3), 620–628. 10.1253/circj.cj-18-0027 29415914

[B36] Wiputra H. Chen C. K. Talbi E. Lim G. L. Soomar S. M. Biswas A. (2018). Human Fetal Hearts with Tetralogy of Fallot Have Altered Fluid Dynamics and Forces. Am. J. Physiology-Heart Circulatory Physiol. 315 (6), H1649–H1659. 10.1152/ajpheart.00235.2018 30216114

[B37] Wiputra H. Lai C. Q. Lim G. L. Heng J. J. W. Guo L. Soomar S. M. (2016). Fluid Mechanics of Human Fetal Right Ventricles from Image-Based Computational Fluid Dynamics Using 4D Clinical Ultrasound Scans. Am. J. Physiology-Heart Circulatory Physiol. 311 (6), H1498–H1508. 10.1152/ajpheart.00400.2016 27663769

[B38] Yalcin H. C. Amindari A. Butcher J. T. Althani A. Yacoub M. (2017). Heart Function and Hemodynamic Analysis for Zebrafish Embryos. Dev. Dyn. 246 (11), 868–880. 10.1002/dvdy.24497 28249360

[B39] Yalcin H. C. Shekhar A. McQuinn T. C. Butcher J. T. (2011). Hemodynamic Patterning of the Avian Atrioventricular Valve. Dev. Dyn. 240 (1), 23–35. 10.1002/dvdy.22512 21181939 PMC3298748

[B40] Yalcin O. T. Ozalp S. S. Tanir H. M. (2002). Assessment of Gestational Trophoblastic Disease by Doppler Ultrasonography. Eur. J. Obstet. Gynecol. Reprod. Biol. 103 (1), 83–87. 10.1016/S0301-2115(02)00026-X 12039472

[B41] Zaidi S. Choi M. Wakimoto H. Ma L. Jiang J. Overton J. D. (2013). De Novo Mutations in Histone-Modifying Genes in Congenital Heart Disease. Nature 498 (7453), 220–223. 10.1038/nature12141 23665959 PMC3706629

[B42] Zebhi B. Wiputra H. Howley L. Cuneo B. Park D. Hoffman H. (2020). Right Ventricle in Hypoplastic Left Heart Syndrome Exhibits Altered Hemodynamics in the Human Fetus. J. Biomech. 112, 110035. 10.1016/j.jbiomech.2020.110035 32971490

